# Evaluating the use of Instagram images color histograms and hashtags sets for automatic image annotation

**DOI:** 10.3389/fdata.2023.1149523

**Published:** 2023-07-04

**Authors:** Stamatios Giannoulakis, Nicolas Tsapatsoulis, Constantinos Djouvas

**Affiliations:** ^1^Department of Communication and Internet Studies, Cyprus University of Technology, Limassol, Cyprus; ^2^Department of Public Communication, Cyprus University of Technology, Limassol, Cyprus

**Keywords:** Instagram hashtags, Instagram images, histogram, Bhattacharyya distance, word embedding, automatic image annotation

## Abstract

Color similarity has been a key feature for content-based image retrieval by contemporary search engines, such as Google. In this study, we compare the visual content information of images, obtained through color histograms, with their corresponding hashtag sets in the case of Instagram posts. In previous studies, we had concluded that less than 25% of Instagram hashtags are related to the actual visual content of the image they accompany. Thus, the use of Instagram images' corresponding hashtags for automatic image annotation is questionable. In this study, we are answering this question through the computational comparison of images' low-level characteristics with the semantic and syntactic information of their corresponding hashtags. The main conclusion of our study on 26 different subjects (concepts) is that color histograms and filtered hashtag sets, although related, should be better seen as a complementary source for image retrieval and automatic image annotation.

## 1. Introduction

Nowadays, online content is generated in an unprecedented pace, including the publication of images on different platforms and fora. Of a paramount importance, however, is the use of efficient and effective techniques that allows the accurate retrieval of images. Image retrieval methods fall within three main categories: text-based image retrieval (TBIR), content-based image retrieval (CBIR), and automatic image annotation (AIA). The text-based techniques are inspired by document retrieval; Keywords are associated with images, e.g., the name of the image file, which are then used as text-based elements against which users' keywords will be matched. CBIR; on the contrary, images are retrieved according to their visual content. Given an example (target) image, CBIR transforms it to a feature vector, which is then used for retrieving images using a similarity metric among their feature vectors. Finally, the AIA approach has as a main idea to automatically learn semantic concept models from a large number of image samples and use these concepts to automatically annotate new images, i.e., assign labels to them. Therefore, AIA can be considered as a combination of TBIR and CBIR because it uses both text-based annotation and content-based image features. AIA is also a method that bridges the semantic gap between low-level image features and high-level semantics (Zhang et al., 2012).

Usually, images are indexed by their visual content based on low-level characteristics, such as color, texture, shape, and spatial layout (Latif et al., 2019). In practice, color as low-level feature is used for image classification and matching because of its effective and low computational cost (Chen et al., 2020). Among them, color histograms (Sergyán, 2008) are quite popular (Takeishi et al., 2018). An advance of color histograms is that are invariant to orientation and scale, and this feature makes it more powerful in image classification. Evidence to the above is the plethora of research studies using color histograms for image retrieval (Liu and Yang, 2013; Theodosiou, 2014; Mufarroha et al., 2020; Zhang et al., 2020).

The previous discussion clearly indicates the importance of efficient and accurate estimation of color-based similarity. In the case of automatic image annotation (AIA), however, images should be annotated with relevant labels. Different approaches exist in the literature for AIA (Tsapatsoulis, 2016, 2020). In this work, we are particularly interested in Instagram hashtags and the possibility of using them for AIA. This is because hashtags have some unique characteristics. They represent a specific topic, or idea, or annotation of an image or text, and they are used regardless of the topic they annotate (Kim and Seo, 2020). In addition, according to Gomez et al. (2020), hashtags is a form of image tagging. Furthermore, they have a metacommunicative use (Daer et al., 2014); The metacommunicative function of hashtags falls into five categories: emphasizing, iterating, critiquing, identifying, and rallying.

Despite their great potentials, only 25% Instagram hashtags are related to the visual content of Instagram images (Giannoulakis and Tsapatsoulis, 2015, 2016b). To alleviate this problem, in our previous study, we proposed different hashtags filtering techniques using classic (Giannoulakis and Tsapatsoulis, 2016a) and sophisticated (Giannoulakis et al., 2017; Giannoulakis and Tsapatsoulis, 2019) methods.

In this study, we attempt to bridge the semantic gap between image low-level features, such as color histogram and high-level semantic content. To do so, we investigated if AIA can be achieved with the aid of Instagram posts, assuming that Instagram is a rich source of implicitly annotated images. More precisely, we evaluate the coherency among similar images' low-level features and their corresponding filtered hashtags, i.e., we assume that similar images (e.g., images containing dogs) should have conceptually similar hashtags. To the best of our knowledge, this is the first research that quantifies the similarity of color histogram and hashtag in Instagram. We show that color histograms and filtered hashtag sets although related should be better seen as complementary source for image retrieval and automatic image annotation.

## 2. Image similarity and Bhattacharyya distance

Image similarly can be calculated using different approaches. Those can be broadly divided into two categories on the basis of the metrics that are used. Intensity-based approaches are based on features (indices) derived from pixel color intensities, while geometry-based approaches use geometric transformations between corresponding pixels (Deza and Deza, 2009; Li and Qian, 2015). In intensity-based similarity computation, the metrics that are usually used are correlation, chi-square, intersection, and Bhattacharyya distance (Arai, 2019; Forero et al., 2019). The geometry-based similarity metrics include pixel correspondence metric, closest distance metric, figure of merit, and partial Hausdorff distance metric (Prieto and Allen, 2003; Li and Qian, 2015). The main drawback of geometry-based similarity metrics is their high computational cost. Thus, intensity-based metrics, usually involving histogram and histogram matching, are frequently adopted.

While a variety of metrics is used for histogram matching, the most common metric is Bhattacharyya (Arai, 2019; Forero et al., 2019) distance, something that is also adopted in this study. Its basic principle is to calculate the distance between two probability distributions *p*(*x*) and *q*(*x*) which are approximated by the corresponding normalized (Equation 3) histogram vectors ${\vec{c}}_{1}$ and ${\vec{c}}_{2}$ (Zhang, 2006; Kayhan and Fekri-Ershad, 2021). Thus, the Bhattacharyya distance between two images *I*_1_ and *I*_2_ with histogram elements *c*_1_(*x*) and *c*_2_(*x*) were computed on a set of color hues X as follows:


(1) $$\begin{array}{l} d\left(I_{1},I_{2}\right)=-ln\left(BC\left({\vec{c}}_{1},{\vec{c}}_{2}\right)\right), \end{array}$$

(2) $$\begin{array}{l} BC\left({\vec{c}}_{1},{\vec{c}}_{2}\right)=\underset{x\in X}{\sum }\sqrt{c_{1}\left(x\right)\cdot c_{2}\left(x\right)}, \end{array}$$

where:


(3) $$\begin{array}{l} \underset{x\in X}{\sum }c_{1}\left(x\right)=1\;\underset{x\in X}{\sum }c_{2}\left(x\right)=1. \end{array}$$

In order to use Bhattacharyya distance as a similarity metric, the reformulation indicated in Equation (4) was applied. It goes without saying, however, that different reformulations expressing a similar logic can be applied.


(4) $$\begin{array}{l} S\left(I_{1},I_{2}\right)=\frac{1}{1+d\left(I_{1},I_{2}\right)} \end{array}$$

It is clear from Equation (4), that the similarity among two image ranges in the (0, 1] interval, with values close to 0 indicating very low similarity, while close to 1 denotes very high similarity (Han, 2015).

Bhattacharyya distance was widely used for computing the low-level content similarity of two images, video frames, or image regions, in a variety of purposes. Chacon-Quesada and Siles-Canales (2017) adopted Bhattacharyya distance as a metric for shot classification of soccer videos. In their effort to develop a moving target tracking algorithm, Ong et al. (2019) used color histograms of frame regions to locate the target object in each frame. Abidi et al. (2017) used histogram of oriented gradients (HoGs) and minimized the Bhattacharyya distance between two sets of gradient orientations expressing the desired and current camera poses, in their vision-based robot control system. Doulah and Sazonov (2017) clustered food-related images using Bhattacharyya similarity. The images were extracted from meal video captured with a wearable camera, and they were indexed using histograms in the HSV color space.

## 3. Word embeddings and Instagram hashtags

In the previous section, we discussed the distance between two histograms as a way to calculate the similarity of two images. In this section, we discuss the second key technology in our study, the word embeddings. Word embeddings were used for finding the similarity of hashtags, focusing on their use in the context of Instagram hashtags.

Word embeddings, i.e., techniques that convert words to numerical vectors retaining semantic and syntactic information, is a state-of-the-art approach in natural language processing, especially in document classification, sentiment analysis (Tsapatsoulis and Djouvas, 2019), and topic modeling (Argyrou et al., 2018; Tsapatsoulis et al., 2022). Word embedding techniques learn the relation between words via training on context examples of each word (Ganguly, 2020) using deep learning methods. Some of the most commonly used word embeddings are GloVe (Gomez et al., 2018), Word2vec (Jiang et al., 2020), and WordRank (Zhang, 2019). Pre-trained word embeddings in a variety of languages are available online, something that boosted their application on an impressive number of different fields.

Weston et al. (2014) used a convolutional neural network to create specific word embeddings for hashtags. The overall aim was to predict hashtags from the text of an Instagram post. Liu and Jansson (2017) tried to identify city events from Instagram posts and hashtags. They used Word2vec embeddings for query expansion, i.e., to identify terms related to the seed posts they used. Hammar et al. (2018) classified Instagram posts text into clothing categories using word embeddings. They used similarity matching via word embeddings to map text to their predefined ontology terms.

Prabowo and Purwarianti (2017) developed a system that helps online shop owners to response to Instagram comments. The system classifies the comments to those that are necessary to answer, those that the online shop owner needs to read, and those to ignore. By comparing the performance of support vector machines (SVMs) and convolutional neural networks (CNNs), they concluded that the combination of word embeddings with CNN learning provides the best combination.

Akbar Septiandri and Wibisono (2017) used Word2vec to detect spam comments on Indonesian Instagram posts. They used the *fastText* library which allows the easy expansion of word matching to short-text (paragraph) matching. Serafimov et al. (2019) proposed hashtag recommendation for online posts using word to paragraph matching with the aid Word2vec vectors. Gomez et al. (2018) combined images and caption to learn the relations between images, words, and neighborhoods, based on Instagram posts related to the city of Barcelona. To achieve their goal they used pretrained Gensim Word2Vec models to discover words that users relate with Barcelona's neighborhoods. Xu et al. (2020) used word embeddings to locate relevant documents in an information filtering system. The researchers produce a topic model tool and trained on users' interest documents. Then, the topic model was applied on incoming documents for estimating the relevance of the new documents to the user.

In the current study, we used Glove (Gomez et al., 2018) pre-trained word embeddings model. Glove is trained on Google News articles and Wikipedia content using the following optimization criterion (Pennington et al., 2014):


(5) $$\begin{array}{l} J=\overset{V}{\underset{i,j=1}{\sum }}f\left(X_{ij}\right){\left(w_{i}^{T}\tilde{w}+b_{i}+{\tilde{b}}_{j}-\log X_{ij}\right)}^{2} \end{array}$$

where *f*(*X*_*ij*_) tabulates the number of times word *j* occurs in the context of word *i*, *w* ∈ ℝ^*d*^ are word vectors, $\tilde{w}\in {\mathbb{R}}^{d}$ are separate context word vectors, *V* is the size of the vocabulary, and *b*_*i*_ is a bias for *w*_*i*_.

## 4. Comparative review

A brief summary of a related study and a comparative review is presented in this section of the study. Zhang et al. (2019) calculated the similarity between brands via posts of brands' followers of Instagram. Image feature extracted using 50-layer ResNet and ImageNet and tags converted into a vector with the help of Word2vec and fasttext^*TM*^. To measure the similarity, they use Pearson correlation and histogram similarity. The study differs from our study due to the different purpose and methodology used. The aim of Zhang et al. was to develop a marketing tool and not to locate the relation between image and hashtags as in our research.

Liu et al. (2015) studied the image color and text similarity in a software application called CITY FEED, that was used for classifying crowd sourced feeds. Image similarity analysis is based on color histogram and computed with Bhattacharyya coefficient. To calculate text similarity, the WordNet algorithm was used. Liu et al.'s research is similar to our research because we also use histogram and Bhattacharyya. However, the nature of the data are totally different. In our research, the data are Instagram photos belonging to a subject/hashtag (e.g., #dog) but are heterogeneous. Data from CITY FEED are not so heterogeneous due to the fact that photos are from the Municipality of Pavia.

## 5. Methodology

The current study was formulated as an experimental study expressed through two null hypotheses using two groups of Instagram posts, the relevant and the irrelevant subset.

*H*0_1_*: In relevant Instagram posts, there is no significant correlation between the similarity of the color histogram of image pairs and the similarity of their corresponding (filtered) hashtags sets*.

*H*0_2_*: There is no significant difference in the average correlation between color histograms and hashtag sets in relevant and irrelevant posts*.

Relevant Instagram posts are posts whose image match the hashtag subject and irrelevant those that their image do not match the hashtag subject (more details about the corpus used in Section 5.2).

At the same time, we expect that the correlation between color histograms and hashtag sets in relevant posts should be significantly higher than that of irrelevant posts.

**Figure 1:**
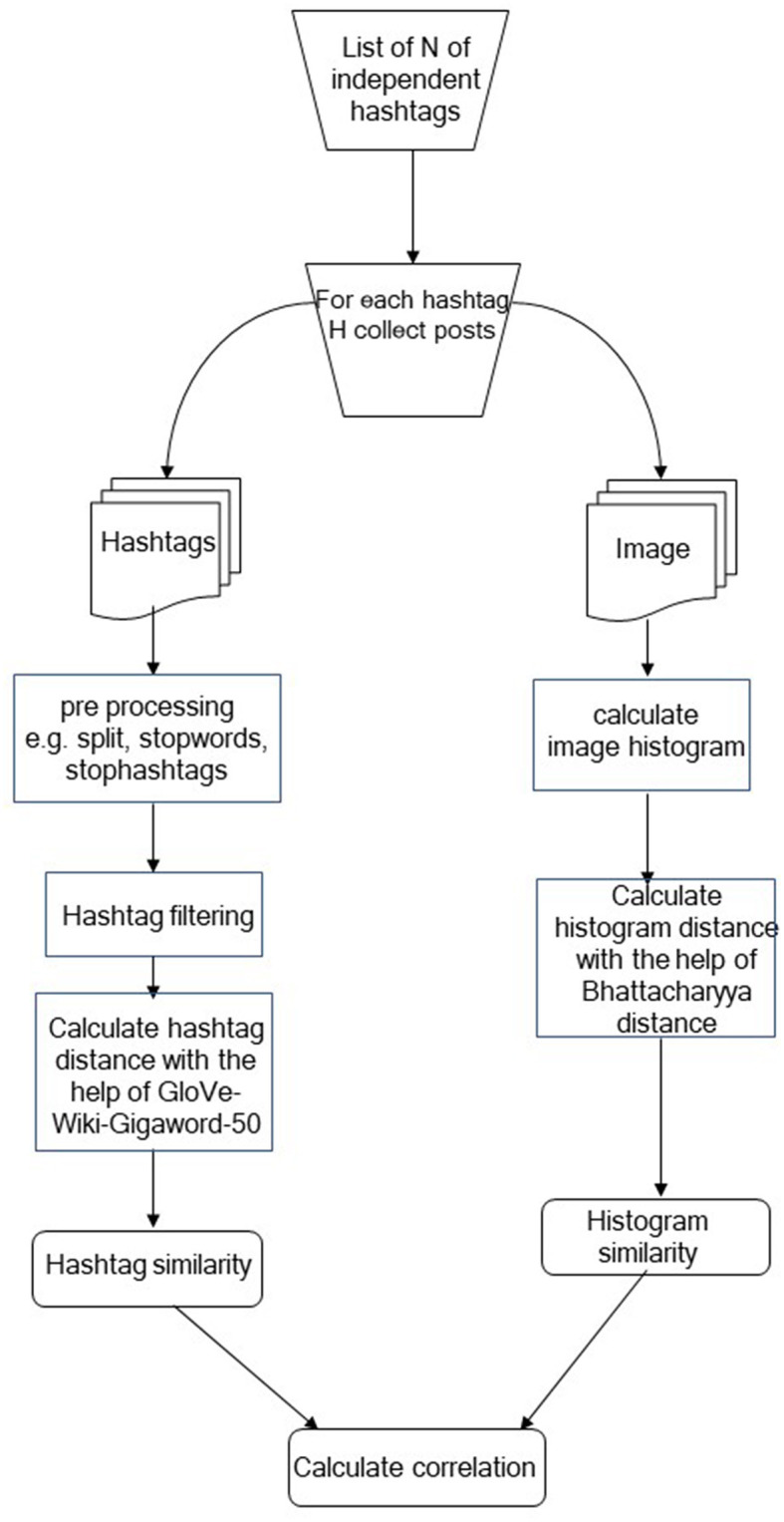
Proposed methodology.

In order to confirm or reject the null hypotheses, the process shown in Figure 1 was followed. First, we selected *N* independent hashtags, which in the context of the current study are referred to as hashtag subjects. For each hashtag subject, we searched Instagram creating two collections of Instagram posts, one containing posts whose image is visually relevant to the subject and one containing posts whose image is not visually relevant to the subject. For posts collected using a hashtag subject, their corresponding image and hashtags were automatically collected using the Beautiful Soup^1^ library of Python. For instance, if we searched Instagram using the hashtag subject #dog, we randomly select posts that depict dog(s) and posts that, despite containing the hashtag #dog, they do not contain a dog. The selection process was random, and only the confirmation regarding the visual relevance is done through human inspection.

For each pair of posts *P*_*i*_ and *P*_*j*_ belonging to the same group, we isolate their corresponding images *I*_*i*_ and *I*_*j*_, and we computed their color histograms, expressing them as vectors ${\vec{c}}_{i}$ and ${\vec{c}}_{j}$, which are then used for computing their Bhattacharyya similarity with the aid of Equation (1). Average similarity scores for both relevant and irrelevant posts were computed for each hashtag subject group of posts as shown in Tables 1, 2, respectively.

**Table 1:** Average Bhattacharyya similarity scores (relevant posts).

	**Bear**	**Cat**	**Chair**	**Dog**	**Dress**	**Elephant**	**Fish**	**Giraffe**	**Guitar**	**Hamster**	**Hat**	**Headband**	**Hedgehog**
Mean	0.722	0.734	0.735	0.749	0.729	0.765	0.668	0.729	0.674	0.744	0.683	0.701	0.718
St. dev.	0.062	0.046	0.062	0.074	0.057	0.042	0.097	0.077	0.054	0.054	0.076	0.072	0.066
Min	0.602	0.654	0.565	0.592	0.613	0.685	0.529	0.602	0.581	0.633	0.533	0.575	0.575
Max	0.840	0.885	0.840	0.885	0.893	0.855	0.885	0.840	0.840	0.840	0.870	0.826	0.840
	**Horse**	**Laptop**	**Lion**	**Mic**	**Monkey**	**Parrot**	**Piano**	**Rabbit**	**Shirt**	**Sunglasses**	**Table**	**Turtle**	**Zebra**
Mean	0.708	0.682	0.767	0.690	0.671	0.693	0.680	0.719	0.699	0.668	0.719	0.706	0.717
St. dev.	0.061	0.043	0.066	0.061	0.096	0.065	0.051	0.055	0.086	0.055	0.044	0.067	0.070
Min	0.592	0.602	0.637	0.565	0.521	0.556	0.592	0.621	0.543	0.565	0.633	0.578	0.606
Max	0.833	0.800	0.893	0.813	0.847	0.800	0.806	0.893	0.870	0.813	0.800	0.826	0.906

**Table 2:** Average Bhattacharyya similarity scores (irrelevant posts).

	**Bear**	**Cat**	**Chair**	**Dog**	**Dress**	**Elephant**	**Fish**	**Giraffe**	**Guitar**	**Hamster**	**Hat**	**Headband**	**Hedgehog**
Mean	0.728	0.690	0.687	0.671	0.631	0.661	0.665	0.717	0.626	0.694	0.664	0.665	0.681
St. dev.	0.074	0.061	0.061	0.067	0.065	0.062	0.092	0.055	0.062	0.079	0.078	0.080	0.071
Min	0.581	0.587	0.580	0.548	0.519	0.555	0.510	0.620	0.523	0.530	0.525	0.518	0.537
Max	0.856	0.821	0.785	0.810	0.754	0.841	0.814	0.846	0.751	0.841	0.798	0.815	0.807
	**Horse**	**Laptop**	**Lion**	**Mic**	**Monkey**	**Parrot**	**Piano**	**Rabbit**	**Shirt**	**Sunglasses**	**Table**	**Turtle**	**Zebra**
Mean	0.706	0.619	0.673	0.620	0.691	0.622	0.707	0.681	0.596	0.686	0.688	0.713	0.694
St. dev.	0.090	0.063	0.080	0.073	0.053	0.079	0.061	0.053	0.062	0.089	0.101	0.077	0.046
Min	0.528	0.526	0.524	0.515	0.614	0.513	0.583	0.562	0.526	0.540	0.538	0.586	0.606
Max	0.889	0.797	0.825	0.820	0.814	0.828	0.824	0.799	0.758	0.809	0.858	0.836	0.795

At the same time, the similarity of their corresponding hashtags, say $H_{i}$ and $H_{j}$, was computed through the process described in Section 5.1. An inherent difficulty, however, derives from the fact that Instagram hashtags are unstructured and ungrammatical, and it is important to use linguistic preprocessing to

1. Remove stophashtags (Giannoulakis and Tsapatsoulis, 2016a), i.e., hashtags that are used to fool the search results of the Instagram platform
2. Split a composite hashtag to its consisting words (e.g., the hashtag “#spoilyourselfthisseason” should be split into four words: “spoil”, “yourself”, “this”, “season”)
3. Remove stopwords that were produced in the previous stage (e.g., the word “this” in the previous example)
4. Perform spelling checks to account for (usually intentionally) misspelled hashtags (e.g., “#headaband”, “#headabandss” should be changed to “#headband”)
5. Perform lemmatization to merge hashtags that share the same or similar meaning
6. Filter out hashtags irrelevant to visual content (Giannoulakis and Tsapatsoulis, 2019).

The aforementioned preprocessing was conducted with the help of the Natural Language ToolKit (NTLK)^2^, Wordnet^3^, and in-house developed code in Python. For each token derived, its corresponding word embeddings vector representation was created using the Genism library (see Section 5.2).

The final step was to compute the correlation between hashtag set (mean) similarities (one hashtag set for each post) and color histogram (mean) similarities with the help of Pearson correlation (Puglisi et al., 2015) coefficient for both the relevant and irrelevant posts. By rejecting the *H*0_1_ null hypothesis, we can conclude that color similarity of images can be predicted by the similarity of their corresponding hashtag sets. Failing to reject the *H*0_1_, null hypothesis indicates that the information obtained from hashtag sets and color histograms, respectively, could be seen either as a complementary source for image retrieval and automatic image annotation or totally uncorrelated. This depends on the decision regarding the second (*H*0_2_) null hypothesis. By rejecting the *H*0_2_ null hypothesis, we can conclude that the information provided by color histograms and hashtag sets is much more correlated in relevant posts (as one would expect from the fact that both have the same, or similar, visual content) than in irrelevant posts.

The purpose of the study is to study the correlation between Instagram image and filtered hashtags sets. The primary purpose of the data collected (Instagram images and hashtags) is exactly to achieve the purpose of this study. Pearson correlation measures the relationship between objects. Moreover, Pearson correlation is among the most commonly used approaches (Puglisi et al., 2015). Building upon the work of Zhang et al. (2019), whom they use Pearson correlation to calculate the similarity between image and tag vectors, for rejecting the *H*0_2_ hypothesis, it is sufficient to compare the correlation coefficient of relevant and irrelevant posts. The methods for comparing coefficient is either Zou's confidence interval or z-score (Diedenhofen and Musch, 2015). In our case, we decided to use z-score, following the approach of other researches (Schreiber et al., 2013; Bhattacharjee et al., 2019; Younes and Reips, 2019).

### 5.1. Matching hashtag sets

Let $H_{i}$ and $H_{j}$ be the filtered hashtag sets of Instagram posts corresponding to the *i*-th and *j*-th Instagram images, respectively. The matching score $R\left(H_{i},H_{j}\right)$ between these two sets is computed as a weighted sum of the pair similarities between the word embeddings of their constituting hashtags, as shown in Equation 6.


(6) $$R({ℋ}_{i},{ℋ}_{j})=\frac{1}{\left|{ℋ}_{i}|\cdot |{ℋ}_{j}\right|}\sum_{h_{ik}\in {ℋ}_{i}}\sum_{h_{j\xi }\in {ℋ}_{j}}cc({\vec{h}}_{ik},{\vec{h}}_{jxi}),$$

where |H| denotes the cardinality of set H, ${\vec{h}}_{ik}$, and ${\vec{h}}_{j\xi }$ are the word embeddings of hashtags *h*_*ik*_ and *h*_*jξ*_ belonging to hashtags sets $H_{i}$ and $H_{j}$ respectively, and *cc*(., .) is the similarity measure used with the word embeddings of Gensim models.^4^

### 5.2. Corpus

**Figure 2:**
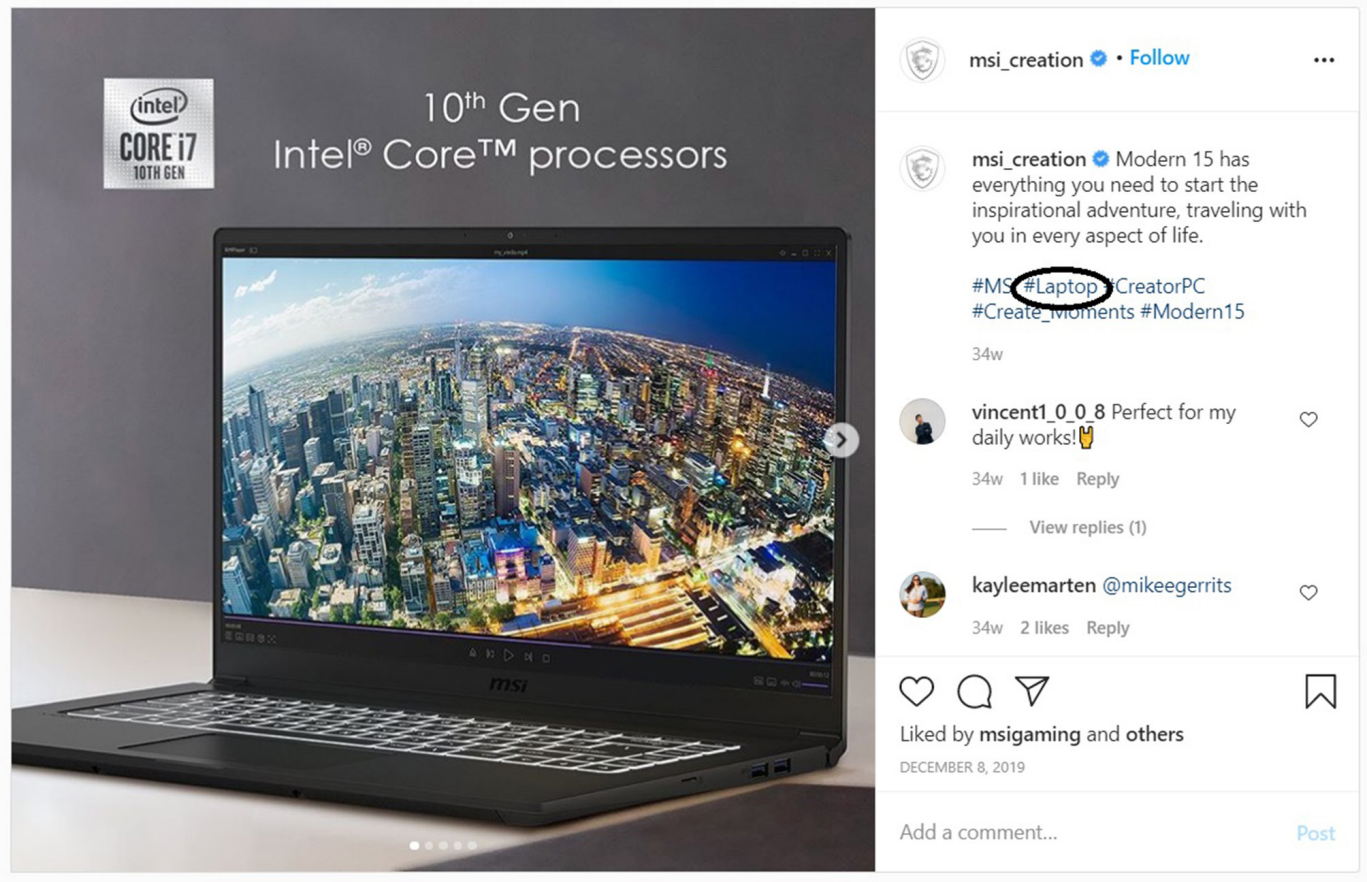
Example of a relevant Instagram post for hashtag #laptop.

**Figure 3:**
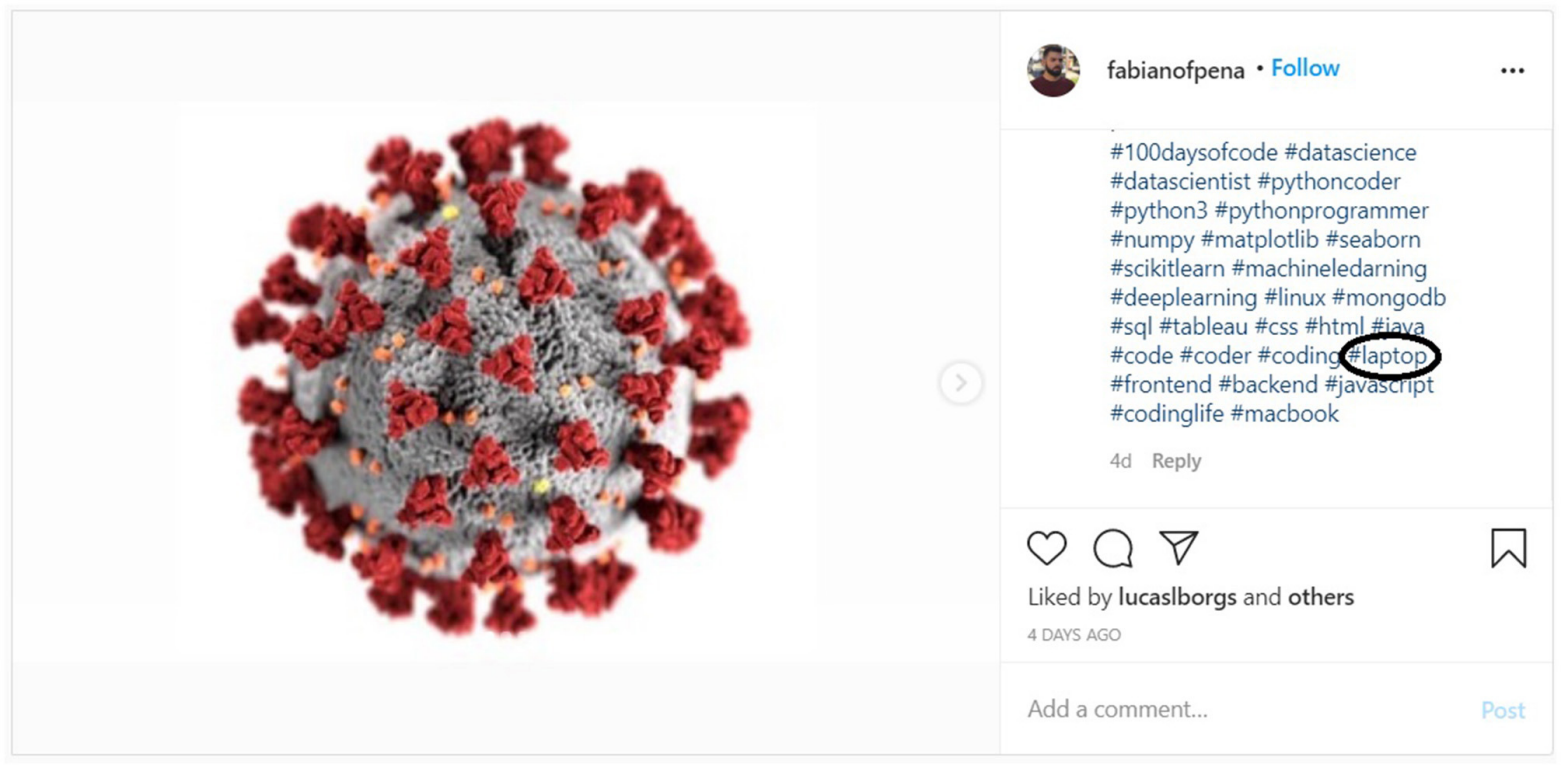
Example of a non-relevant Instagram post for hashtag #laptop.

To evaluate the proposed methodology, along with the two null hypotheses, we randomly selected 26 independent hashtag subjects.^5^ For each one of the 26 hashtag subjects, we collected 10 relevant and 10 irrelevant Instagram posts (images and corresponding hashtags). The above process created a corpus of 520 (260 relevant and 260 non-relevant) images and 8199 hashtags (2883 for relevant images and 5316 non-relevant images). As already mentioned, relevant Instagram posts are posts whose image match the hashtag subject, and irrelevant posts are those that their image do not match the searched hashtag. Figures 2, 3 show an example of a relevant and irrelevant Instagram post, respectively, for the hashtag subject #laptop.

## 6. Experimental results and discussion

In the previous section, we presented the methodology adopted, along with the similarities calculated for images (Table 1 for relevant posts and Table 2 for irrelevant post) and hashtags (Table 3 for relevant posts and Table 4 for irrelevant post). In this section, we utilized the aforementioned results for producing aggregates, which in turn will be used for accept or reject the two null hypotheses. In order to calculate the Bhattacharyya distance and filtered hashtag similarity, we used the Python OpenCV^6^ and Gensim libraries, respectively.

**Table 3:** Average similarity of hashtag sets (relevant posts).

	**Bear**	**Cat**	**Chair**	**Dog**	**Dress**	**Elephant**	**Fish**	**Giraffe**	**Guitar**	**Hamster**	**Hat**	**Headband**	**Hedgehog**
Mean	0.350	0.255	0.263	0.224	0.250	0.247	0.167	0.197	0.254	0.257	0.294	0.189	0.160
St. dev.	0.084	0.137	0.109	0.097	0.068	0.115	0.075	0.047	0.083	0.143	0.110	0.043	0.084
Min	0.183	0.049	0.103	0.097	0.107	0.100	0.069	0.126	0.148	0.087	0.126	0.124	0.046
Max	0.575	0.781	0.560	0.471	0.404	0.643	0.356	0.316	0.534	0.920	0.633	0.297	0.457
	**Horse**	**Laptop**	**Lion**	**Mic**	**Monkey**	**Parrot**	**Piano**	**Rabbit**	**Shirt**	**Sunglasses**	**Table**	**Turtle**	**Zebra**
Mean	0.220	0.257	0.225	0.220	0.251	0.151	0.217	0.278	0.233	0.251	0.258	0.241	0.255
St. dev.	0.117	0.042	0.050	0.043	0.090	0.069	0.079	0.168	0.052	0.083	0.070	0.067	0.077
Min	0.059	0.162	0.137	0.114	0.044	0.043	0.090	0.080	0.140	0.138	0.109	0.133	0.130
Max	0.692	0.337	0.363	0.310	0.445	0.306	0.404	0.946	0.357	0.553	0.413	0.395	0.483

**Table 4:** Average similarity of hashtag sets (irrelevant posts).

	**Bear**	**Cat**	**Chair**	**Dog**	**Dress**	**Elephant**	**Fish**	**Giraffe**	**Guitar**	**Hamster**	**Hat**	**Headband**	**Hedgehog**
Mean	0.148	0.134	0.183	0.209	0.240	0.189	0.138	0.139	0.238	0.114	0.261	0.187	0.158
St. dev.	0.062	0.072	0.094	0.068	0.115	0.078	0.078	0.062	0.118	0.058	0.060	0.034	0.059
Min	0.051	0.030	0.024	0.103	0.064	0.094	0.019	0.053	0.068	0.035	0.163	0.120	0.052
Max	0.322	0.291	0.395	0.330	0.464	0.507	0.332	0.291	0.519	0.298	0.417	0.249	0.277
	**Horse**	**Laptop**	**Lion**	**Mic**	**Monkey**	**Parrot**	**Piano**	**Rabbit**	**Shirt**	**Sunglasses**	**Table**	**Turtle**	**Zebra**
Mean	0.094	0.161	0.210	0.187	0.121	0.102	0.178	0.181	0.164	0.104	0.178	0.143	0.119
St. dev.	0.056	0.073	0.059	0.048	0.077	0.051	0.083	0.076	0.066	0.038	0.076	0.040	0.052
Min	0.013	0.028	0.115	0.107	0.017	0.015	0.054	0.035	0.081	0.043	0.065	0.058	0.020
Max	0.206	0.300	0.361	0.303	0.274	0.257	0.388	0.352	0.367	0.178	0.336	0.220	0.241

**Figure 4:**
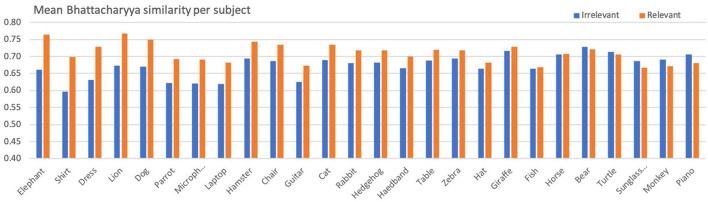
Mean Bhattacharyya similarity per subject for relevant and irrelevant posts.

Table 5 presents the aggregated statistics (across all subjects) regarding the color histogram similarities for relevant and irrelevant posts, while Figure 4 depicts a more detailed representation per subject. Despite the fluctuations across various subjects, the aggregated mean for color histogram similarity in relevant posts is significantly higher than that of irrelevant posts (*t* = 4.35, *p* < 0.01, *df* = 25, *d* = 1.22).^7^

**Table 5:** Aggregated statistics of Bhattacharyya similarity across all subjects for relevant and irrelevant posts.

	**Mean**	**St. dev**.	**Min**	**Max**
Relevant images	0.710	0.028	0.668	0.767
Non-relevant images	0.672	0.034	0.596	0.728

**Figure 5:**
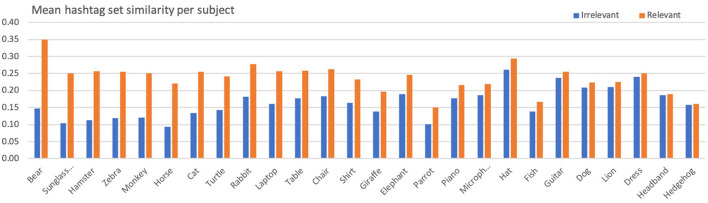
Mean hashtag sets similarity per subject for relevant and irrelevant posts.

Similarly to the above, Table 6 shows the aggregated statistics (across all subjects) regarding the hashtag sets similarities for relevant and irrelevant posts. At the same time, Figure 5 presents a per subject comparison. Fluctuations across various subjects also appear here, as in the case of color histogram similarities. Once again, the aggregated mean for hashtag sets similarity in relevant posts is significantly higher than that of irrelevant posts (*t* = 6.04, *p* < 0.01, *df* = 25, *d* = 1.71).

**Table 6:** Aggregated statistics of hashtag sets similarity across all subjects for relevant and irrelevant posts.

	**Mean**	**St. dev**.	**Min**	**Max**
Relevant images	0.237	0.041	0.151	0.350
Non-relevant images	0.165	0.043	0.094	0.261

**Figure 6:**
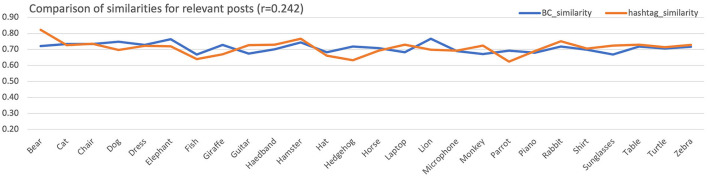
Mean similarities across all subjects for the color histogram and the hashtags sets for the relevant posts. For better visualization, equalization of aggregated means has been performed.

Figure 6 depicts the comparison^8^ of mean similarity between filtered hashtag sets and color histograms for the relevant posts. The Pearson correlation is *r*_*r*_ = 0.242, which is lower than the critical value *r*_*c*_ = 0.33 obtained for *df* = 24, and its level of significance *a* = 0.05. Thus, the *H*0_1_ null hypothesis, that the similarity of color histograms and filtered hashtags sets in the relevant posts are significantly correlated, cannot be rejected.

**Figure 7:**
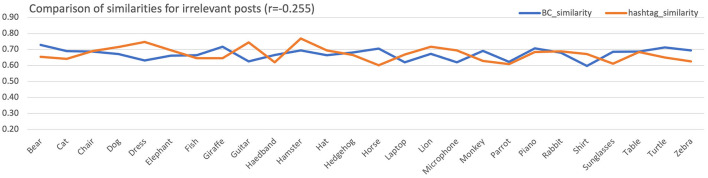
Mean similarities across all subjects for the color histogram and the hashtags sets for the irrelevant posts. For better visualization, equalization of aggregated means has been performed.

Figure 7 examines the case of irrelevant posts. The Pearson correlation in this case is *r*_*i*_ = −0.255, showing that in irrelevant posts information obtained through the color histograms and the hashtags sets are contradicting. This was expected since the irrelevant datasets consisted of posts whose visual content did not match the hashtag subject.

In order to examine the second null hypothesis (*H*0_2_), we have to compare two correlation coefficients and check the significance of their difference, assuming a normal distribution.

The z-score of a correlation coefficient is obtained using the following formula (Yuan et al., 2013):


(7) $$\begin{array}{l} z_{k}=0.5\cdot log\left(\frac{1+r_{k}}{1-r_{k}}\right). \end{array}$$

Applying formula 7, we get *z*_*r*_ = 0.247 and *z*_*i*_ = −261. In order to test the significance of z-score difference, we need to normalize the standard error (Equation 9):


(8) $$\begin{array}{l} z=\frac{z_{r}-z_{i}}{\sigma_{z_{r}-z_{i}}} \end{array}$$

where


(9) $$\begin{array}{l} \sigma_{z_{r}-z_{i}}=\sqrt{\frac{1}{n_{r}-3}+\frac{1}{n_{i}-3}} \end{array}$$

Given that *n*_*r*_ = *n*_*i*_ = 26 (the number of subjects) from Equation (8) and (9), we get *z* = 1.73, which gives *p* = 0.042. Thus, the *H*0_2_ null hypothesis is rejected at a significant level *a* = 0.05.

## 7. Conclusion

The purpose of the study was to examine if can bridge the gap between low-level features and high-level semantic content. To achieve the aforementioned purpose, we calculated the correlation between color similarity of Instagram 26 images and their corresponding filtered hashtag sets. While no statistically significant correlation between the color histogram and the corresponding hashtag sets similarities was found, the hashtags can be seen as a complementary source for image retrieval and automatic image annotation. This is supported by the fact that the correlation difference between the similarity of color histograms and corresponding hashtag sets in relevant and irrelevant posts is both high and significant. This means that Instagram images of similar visual content, i.e., relevant posts, share similar hashtags as well. This is not the case for Instagram images of varying visual content that share few (at least one) hashtags.

Another finding of this study is that both the color histogram and the hashtag sets similarities are significantly higher in relevant posts than in irrelevant ones. Thus, it is confirmed that both color histograms and hashtag sets provide important information related to the visual content of Instagram images. Comparing the effect size using the Cohen *d* coefficient for color histograms and hashtags sets, one can observe that hashtag sets provide more rich information regarding the visual content of Instagram images.

Finally, we should state that we recognize that the use of color histograms might introduce some limitations to this study due to their inherent limitations (it ignores spatial relationships between pixels, it is sensitive to changes in illumination, it does not take into account the semantic meaning of the objects or scenes, and it is susceptible to noise and occlusion in images). However, we do not believe that those limitations were sufficient for changing the overall findings of this study, especially for the irrelevant posts dataset since images of the same group were irrelevant. Nevertheless, in future work, we will use different images' representations along with more images, alleviating any possible statistical bias attributed to either the images' representation or the images used. Furthermore, we plan to investigate experimentally whether the information provided by color histograms and hashtags sets is, indeed, complementary in the context of image retrieval. Our assumption is that a hybrid retrieval scheme that combines color histograms and hashtag sets will give significantly better retrieval results than each one of the individual methods alone. Finally, in an attempt to improve the correlation among the color histogram and the corresponding hashtag sets similarities, a more sophisticated filtering technique extending our previous study Giannoulakis and Tsapatsoulis (2019) will be considered.

## Data availability statement

The raw data supporting the conclusions of this article will be made available by the authors, without undue reservation.

## Author contributions

All authors listed have made a substantial, direct, and intellectual contribution to the work and approved it for publication.

## Footnotes

^1^ http://www.crummy.com/software/BeautifulSoup/bs4/doc/

^2^ https://www.nltk.org/

^3^ https://wordnet.princeton.edu/

^4^ https://radimrehurek.com/gensim/models/word2vec.html

^5^List of hashtag subjects selected: #bear, #cat, #chair, #dog, #dress, #elephant, #fish, #giraffe, #guitar, #hamster, #hat, #headband, #hedgehog, #horse, #laptop, #lion, #mic, #monkey, #parrot, #piano, #rabbit, #shirt, #sunglasses, #table, #turtle, #zebra.

^6^ https://opencv.org/

^7^*d* is the Cohen coefficient denoting the effect size.

^8^Equalization of means has been performed for better visualization.
